# Renal pseudoaneurysm formation post allograft biopsy: a case report

**DOI:** 10.1259/bjrcr.20150502

**Published:** 2016-12-23

**Authors:** Samir G Mallat, Rima Abou Arkoub, Bassam El Achkar, Charbel Saade, Fadi El-Merhi

**Affiliations:** ^1^Department of Internal Medicine, American University of Beirut Medical Center, Beirut, Lebanon; ^2^Department of Diagnostic Radiology, American University of Beirut Medical Center, Beirut, Lebanon

## Abstract

Renal pseudoaneurysm (PSA) is a rare complication post kidney transplant biopsy that accounts for less than 1% of allograft dysfunction. Imaging guidelines in the diagnosis of renal PSA have not yet been developed owing to the low occurrence and limited data availability. However, contrast-enhanced CT and magnetic resonance angiography (MRA) are the preferred modalities in detecting PSA owing to the high contrast and spatial resolution. However, magnetic resonance angiography is preferred since non-contrast imaging techniques can see blood flow patterns in renal PSA without the use of contrast media that may alter renal function. We present a rare complication in a 48-year-old male receiving a living related kidney transplant and found to have renal PSA post allograft biopsy. We review the clinical features, imaging and treatment outcome with the developed PSA in the transplanted kidney post allograft biopsy.

## Background

Renal core biopsy remains the “gold standard” for the diagnosis of renal allograft dysfunction. The rate of transplant renal biopsy complications ranges between 0.06 and 13%.^1–6^ Arterial injuries, mainly haemorrhage, are the predominant complication related to transplant kidney biopsy; the rate of frank and occult haematuria secondary to renal biopsy has been reported to be between 5 and 40%.^7,8^ Other arterial injuries include arteriovenous fistula (AVF) or PSA. A PSA results from an injury to one or more layers of the arterial wall, and its incidence following percutaneous biopsy was estimated to be 5%.^9^ PSA is a serious condition because the arterial perforation is occluded solely by haematoma and connective tissue. It can easily become a life-threatening haemorrhage if the balance between the intraluminal hydrostatic pressure and the tamponade effect of the surrounding haematoma and connective tissue changes. We present a 48-year-old patient who underwent a renal biopsy following renal transplant that was complicated with a PSA.

Institutional Review Board approval was obtained before any step of this case report.

## Case presentation

A 48-year male with a history of end-stage renal disease secondary to focal segmental glomerulosclerosis received a kidney transplant from a living related donor in 2005 after 4 years of haemodialysis. Post transplant, the patient was placed on triple immunosuppressive therapy consisting of cyclosporine A, steroids and mycophenolate mofetil.

In 2011, routine laboratory workup showed an increase in serum creatinine. This was followed by renal allograft biopsy, which revealed evidence of cyclosporine nephrotoxicity. Cyclosporine A was stopped, and the patient was maintained on prednisone and mycophenolate mofetil. A progressive decrease in his serum creatinine level, back to its baseline value of 2.0 mg%, was then noted.

In 2012, the patient presented to our centre complaining of fatigue, nausea and decreased appetite of 10 days duration. Physical examination was unremarkable except for a bruit over the transplanted kidney in the right iliac fossa. Laboratory results revealed a haemoglobin concentration of 9.8 g l^–1^, serum creatinine level of 6.1 mg% and estimated Glomelular Filtration Rate (eGFR) by Modification of Diet in Renal Disease (MDRD) formula of 10 ml min^–1 ^ 1.73 m^–2^. Urine analysis was normal.

**Figure 1.**
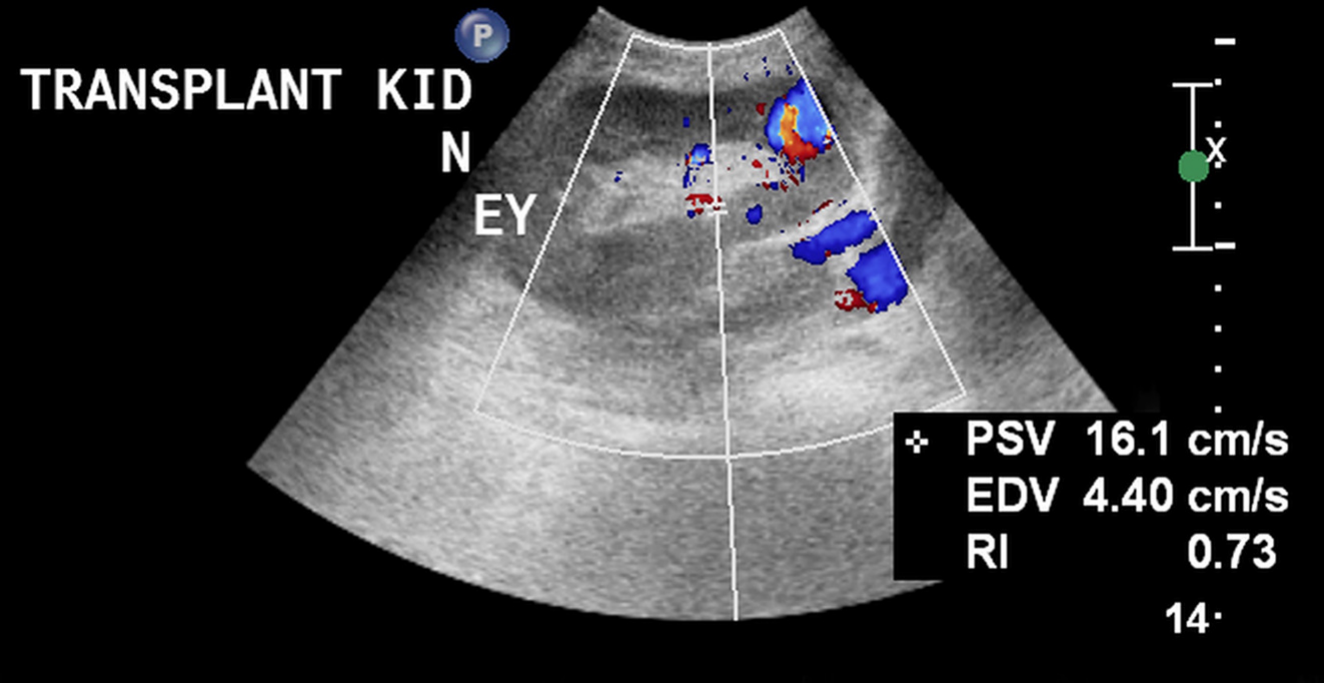
Doppler ultrasound showing the typical “ying-yang” sign that is highly suggestive of renal pseudoaneurysm.

**Figure 2.**
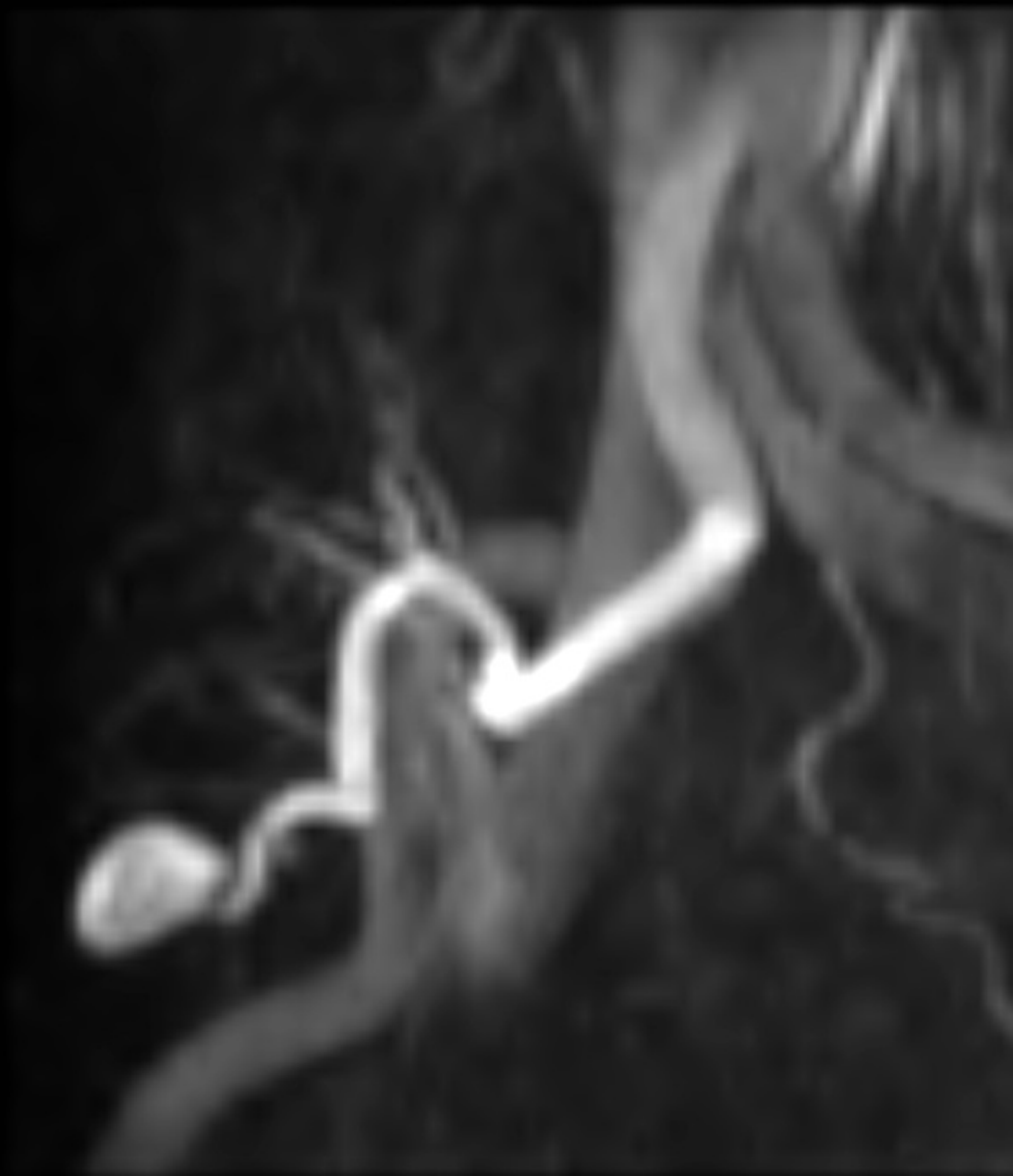
Magnetic resonance angiography of the transplanted kidney showing the location of the pseudoaneurysm that is supplied by the lower lobar artery.

Ultrasound Doppler study of the transplanted kidney was performed, which revealed a normal-sized kidney with normal corticomedullary differentiation, with its lower pole demonstrating a cystic structure measuring 1.7 cm. Colour Doppler imaging showed the typical “ying-yang” sign that was highly suggestive of PSA (Figure 1). Magnetic resonance angiography (MRA) without gadolinium was performed to further confirm that the 1.4 × 1.7 cm PSA was located in the lower pole of the transplanted kidney and was supplied by the lower lobar artery (Figure 2). Because of the size of the PSA and the risk of rupture, a decision to embolize the PSA was taken in a multidisciplinary meeting and informed consent was obtained.

**Figure 3.**
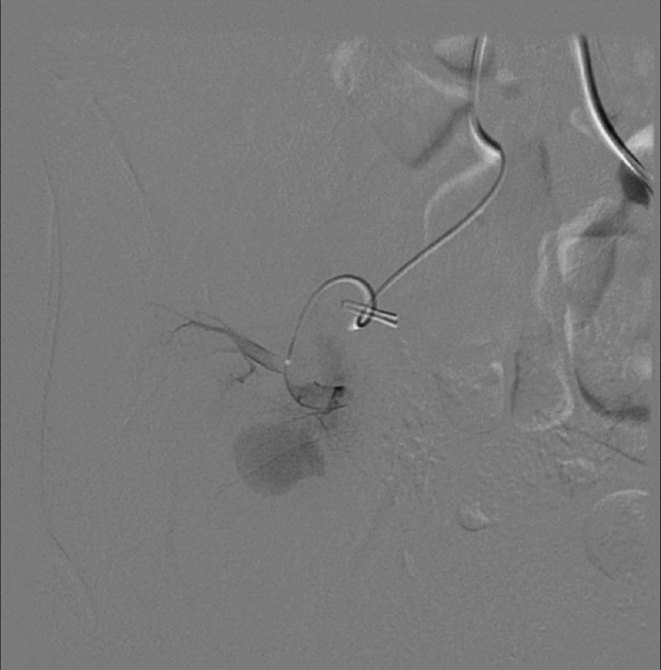
Conventional angiogram of the transplanted right kidney demonstrates selective catheterization and coil embolization of the lobular artery supplying the pseudoaneurysm.

**Figure 4.**
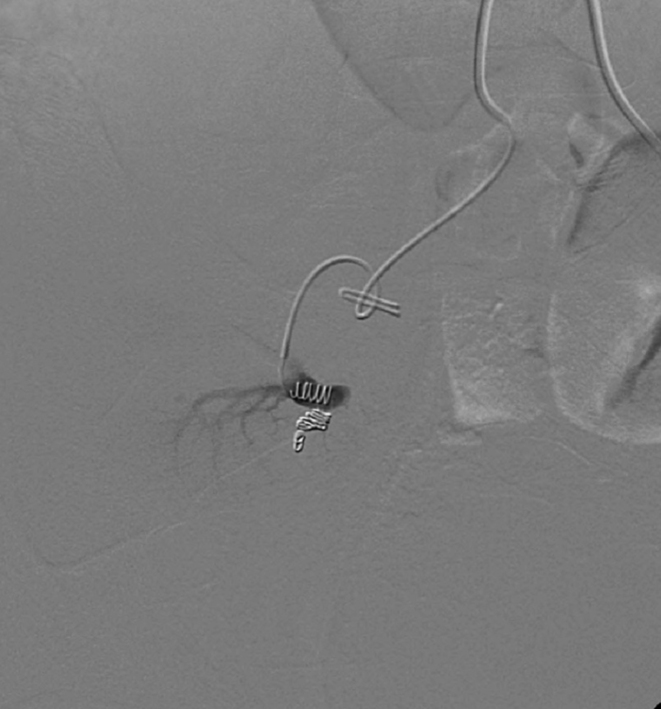
Conventional angiogram of the transplanted right kidney demonstrates selective cauterization of the lobular artery supplying the pseudoaneurysm.

**Figure 5.**
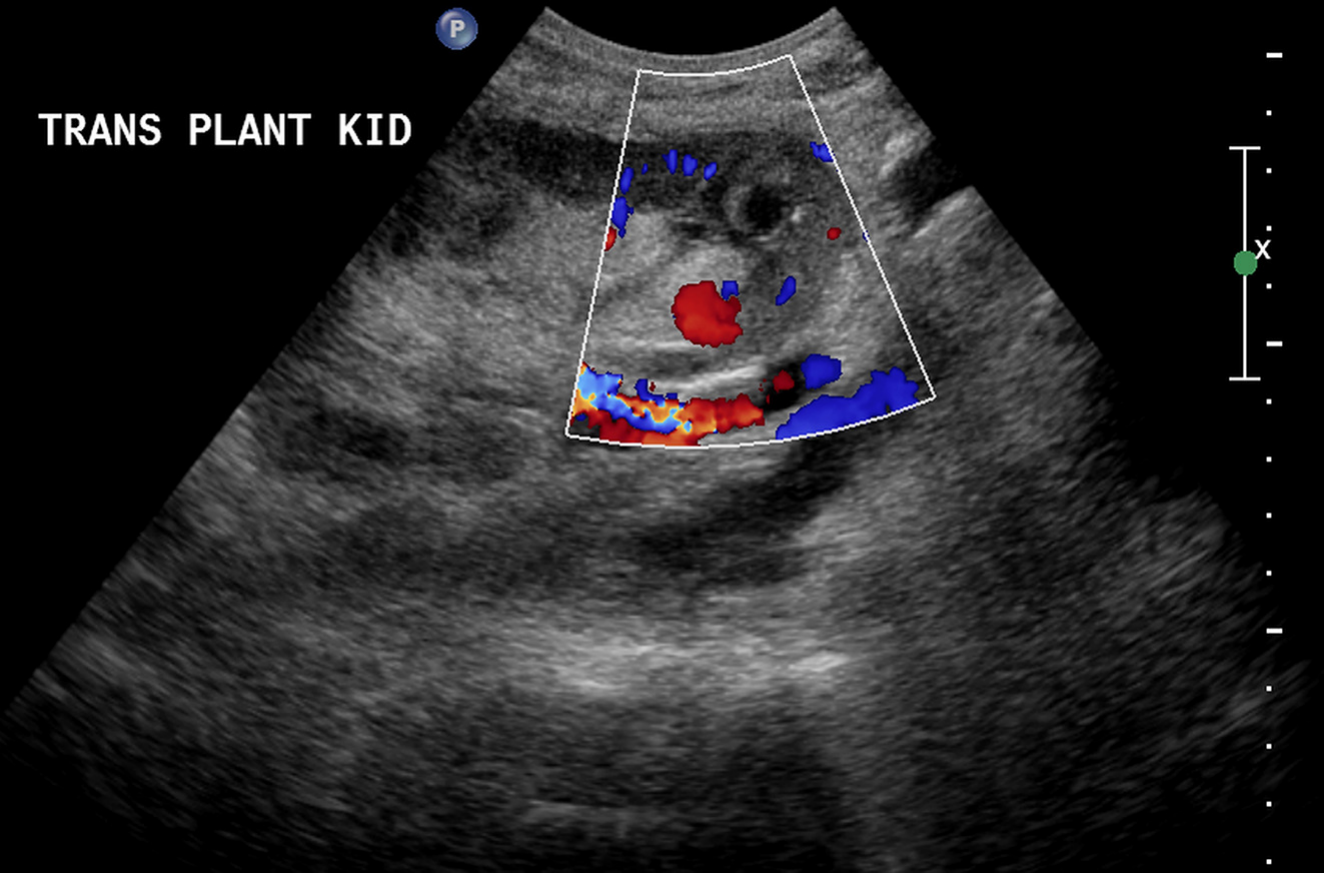
Doppler ultrasound of the transplanted kidney showing a decrease in the size of the pseudoaneurysm with no detectable flow.

Intervention consisted of a selective right common iliac artery angiography using a 5 Fr cobra catheter that delineated the transplanted kidney PSA. Using a 3 Fr Progreat catheter, the single arterial supply for the PSA was selected. Two Tornado coils (one 2 × 3 mm, and one 3 × 4 mm) and two Nester coils (3 × 3 mm) were deployed (Figure 3). Contrast injection immediately post procedure confirmed the absence of flow to the PSA (Figure 4). The next day, a repeat ultrasound was performed and showed a decrease in the size of the PSA with no detectable flow (Figure 5). The patient’s serum creatinine level improved progressively to reach its baseline value over a few days.

## Discussion

Vascular complications requiring intervention account for 0.2–2% of graft biopsy-related injuries.^10,11^ AVF and PSA are the most common iatrogenic biopsy-related vascular injuries in native and transplanted kidneys. However, PSAs following renal transplantation are rare, with an incidence rate of less than 1%.^12^ In general, they may form at the arterial anastomosis,^13^ occur after renal biopsy^14^ or may be due to a mycotic infection.^15^

The pathogenesis could be related to haemorrhage triggered by biopsy-induced damage to the arterial wall, and in the course of repair, the endothelium is dissolved leaving a sac in continuity with the endoluminal space.^16^ The vessels involved include any of the segmental or arcuate ones.^17^

Signs and symptoms are variable and include evidence of infection near the graft, massive or prolonged haematuria or a bruit over the graft.^18^ Deterioration of the renal function after transplant kidney biopsies has been occasionally reported.^19^ In our patient, the increasing intracapsular pressure due to an expanding aneurysm may have contributed to the declining graft function through occlusion of the capillary bed.

Imaging techniques applicable to the diagnosis of PSA include enhanced CT, duplex Doppler sonography, colour Doppler sonography and angiography. Owing to its high availability, speed and high resolution, enhanced CT scan is the initial modality of choice for the diagnosis of PSA. However, owing to the increased level of creatinine and renal failure in our patient, contrast CT was avoided in order not to worsen the problem. Scintigraphy can be used to detect some large PSAs.^17^ The Doppler findings of PSA show highly turbulent pulsatile flow in their central lumen with classic “machinelike to-and-fro flow at their neck, with disorganized colour flow within an apparent cyst, it has the same surrounding parenchymal vibration colour as AVF flow.”^17^ Angiography is considered the gold standard technique for the detection of AVFs and PSA, which appears as an extravascular spherical collection of contrast material.^17^ This case illustrates that transcatheter embolization is an effective endovascular technique to treat biopsy-related renal allograft vascular injury; embolization can be performed superselectively using a micro-catheter and an embolic agent micro-coils in most cases.^20^ Superselective embolization performed with a coaxial catheter and metallic coils minimizes the loss of functioning allograft tissue. It also allows the occlusion of targeted vessels in a precise and definitive manner, unlike embolization with particles that may reflux into non-targeted branches.^21^

## Conclusions

PSA is a rare but serious complication following percutaneous renal biopsy. A high index of suspicion should be prompted by symptoms and be followed by imaging for diagnosis. Treatment and complete reversibility of most renal PSA arising from small vessels can be achieved using a minimally invasive small transcatheter embolization technique.

## Learning points

1. Transcatheter embolization, an effective endovascular technique to treat biopsy-related renal allograft vascular injury, allows the occlusion of targeted vessels in a precise and definitive manner, unlike embolization with particles that may reflux into non-targeted branches.
2. The intervention consisted of a selective right common iliac artery angiography using a 5 Fr cobra catheter.
3. Contrast injection immediately post procedure confirmed the absence of flow to the PSA.
4. A repeat ultrasound was performed and showed a decrease in the size of the PSA with no detectable flow.
5. The patient’s serum creatinine improved progressively to reach its baseline value over a few days.

## Consent

Informed consent to publish this case was obtained and is held on record.
